# Good vibrations: tactile cueing for freezing of gait in Parkinson’s disease

**DOI:** 10.1007/s00415-023-11663-9

**Published:** 2023-03-21

**Authors:** E. C. Klaver, J. P. P. van Vugt, B. R. Bloem, R. J. A. van Wezel, J. Nonnekes, M. C. Tjepkema-Cloostermans

**Affiliations:** 1 Department of Neurology and Clinical Neurophysiology, Medical Spectrum Twente, Enschede, The Netherlands; 2 Department of Biophysics, Donders Institute for Brain, Cognition and Behaviour, Radboud University, Heyendaalseweg 135, P.O. Box 9102, 6525 AJ Nijmegen, The Netherlands; 3 Department of Neurology, Donders Institute for Brain, Cognition and Behaviour, Centre of Expertise for Parkinson and Movement Disorders, Radboud University Medical Centre, Nijmegen, The Netherlands; 4 Department of Rehabilitation, Donders Institute for Brain, Cognition and Behaviour, Centre of Expertise for Parkinson and Movement Disorders, Radboud University Medical Centre, Nijmegen, The Netherlands; 5 Department of Biomedical Signals and Systems, MedTech Centre, University of Twente, Enschede, The Netherlands; 6 Department of Rehabilitation, Sint Maartenskliniek, Nijmegen, The Netherlands; 7 MedTech Centre, Clinical Neurophysiology, University of Twente, Enschede, The Netherlands

**Keywords:** Cues, Parkinson’s disease, Gait disorders/neurologic, Parkinson’s disease/therapy*, Haptic feedback technology, Feedback, Wearable electronic devices*

## Abstract

**Background:**

Cueing strategies can alleviate freezing of gait (FOG) in people with Parkinson’s disease (PD). We evaluated tactile cueing delivered via vibrating socks, which has the benefit of not being noticeable to bystanders.

**Objective:**

To evaluate the effect of tactile cueing compared to auditory cueing on FOG.

**Methods:**

Thirty-one persons with PD with FOG performed gait tasks during both ON and OFF state. The effect of open loop and closed loop tactile cueing, as delivered by vibrating socks, was compared to an active control group (auditory cueing) and to a baseline condition (uncued gait). These four conditions were balanced between subjects. Gait tasks were videotaped and annotated for FOG by two experienced raters. Motion data were collected to analyze spatiotemporal gait parameters. Responders were defined as manifesting a relative reduction of > 10% in the percent time frozen compared to uncued gait.

**Results:**

The average percent time frozen during uncued gait was 11.2% in ON and 21.5% in OFF state. None of the three tested cueing modalities affected the percentage of time frozen in either the ON (*p* = 0.20) or OFF state (*p* = 0.12). The number of FOG episodes and spatiotemporal gait parameters were also not affected. We found that 22 out of 31 subjects responded to cueing, the response to the three types of cueing was highly individual.

**Conclusions:**

Cueing did not improve FOG at the group level; however, tactile as well as auditory cueing improved FOG in many individuals. This highlights the need for a personalized approach when using cueing to treat FOG.

## Introduction

Freezing of gait (FOG) is a debilitating symptom of Parkinson disease (PD) [1], described as having a feeling of the feet being ‘glued to the floor’ [2]. FOG occurs in 20–60% of persons in later stages of PD [3]. It highly impairs mobility, causes falls and reduces the quality of life [2]. FOG improves only partially in response to pharmacological treatment; therefore, non-pharmacological treatments, such as external cueing, are a key complementary treatment modality [4–6]. External cues include rhythmic temporal or spatial stimuli that can help to initiate or to continue movements, such as gait [5, 7]. Visual and auditory cueing effectively reduce FOG [8–11]. The response to cueing, however, varies greatly between people with PD [6, 12]; indeed, not all persons with PD respond to cueing, and for some, gait may even worsen [4]. Although the exact working mechanism of various types of cueing remains unknown, this specific compensation strategy aims to shift the habitual (internally driven) motor control of a person with PD to a more goal-directed (externally driven) control. In very practical terms, this means that people with FOG can overcome their FOG episodes by focusing their attention on gait via the external cues [5, 13]. Just like the more widely studied auditory and visual cues, tactile cues act in the same way to draw a person’s attention to the act of walking.

Alongside their positive effects, auditory and visual cueing both also have practical issues. Auditory cueing requires people to wear an earbud to provide the cueing, or the cueing is provided by a loudspeaker, which in both circumstances may hamper the communication with bystanders or interfere with other relevant auditory input from the environment. Visual cueing (e.g. by projecting a laser beam onto the floor) is also noticeable to bystanders and requires the end-user to look at their feet while walking, which can result in a poorer posture. Given these limitations, it remains difficult to translate visual and auditory cueing strategies into an ambulatory device that is effective, but at the same time also socially acceptable [14].

Vibrotactile cueing could overcome these limitations of visual and auditory cueing. Indeed, several smaller studies reported that vibrotactile cueing afforded a significant reduction in the percentage of time spent frozen and the number of freezing episodes by [15–17]. However, the efficacy of tactile cueing on gait improvement in larger cohorts remains unclear. A previous study included 43 people with PD and found positive effects of tactile cueing on FOG during turning [18]. The effects during gait remain to be investigated.

Our group previously reported a person with PD who experienced a marked improvement of severe FOG by tactile cueing delivered via vibrating socks [14]. We hypothesize that drawing a person’s attention to the feet—which are essential for gait—will result in effective tactile cueing. An additional benefit of including tactile cueing in a sock is that in practice it can be worn underneath clothing, making it socially acceptable. In the current study, we evaluate the effect of these vibrating socks in a larger cohort using a within-subject design. We evaluate two versions of tactile cueing: closed loop and open loop tactile cueing. Closed loop cueing provides a flexibly timed tactile cue that occurs only when the cued foot of the participant reaches the stance phase of the gait cycle. Open loop stimulation provides tactile cueing to a predetermined, fixed frequency, which is matched with the participants’ preferred cadence. We compared the effect of both stimulation modes, as delivered by the vibrating socks, to an active control group (auditory cueing) and to a baseline condition (uncued gait). We hypothesized that tactile cueing would decrease the duration and number of FOG episodes and also improve spatiotemporal gait parameters compared to uncued gait. We expected the effect of the vibrating socks to be comparable to that of auditory cueing.

## Methods

### Participant selection

We included 40 people with PD and a recent history of disabling or regular FOG. Disabling or regular FOG was defined as presence of FOG several times a day during the past month and lasting longer than 1 s. Importantly, FOG presence had to be verified objectively by an experienced neurologist. Exclusion criteria were gait impairments due to any factor other than PD, sensory impairments of the lower legs hampering perception of the vibrating socks, and cognitive impairment that hampered participants in understanding the research purpose or accompanying instructions. Participants were screened for gnostic sensory impairments prior to inclusion in two different ways. If participants had a routinely scheduled appointment, the socks were applied. If they were able to perceive the vibrations of the socks in both feet, they were included. Participants who did not have a routinely scheduled appointment were asked to test their gnostic sensibility at home. To this aim, the caregivers of the participants were instructed to gently stroke the sole of the feet of the participant with a cotton swab. If the cotton swab was not perceived, they were excluded from participation. The study was approved by the local ethical committee (trial ID NL68729.044.19) and registered in the Dutch trial registry (NL7679). All participants gave written informed consent to participate in the study.

### Measurement protocol

Subjects participated during two sessions on two separate days, one in their ON dopaminergic state and one in the OFF dopaminergic state. FOG is most common and also most pronounced in people with PD in the OFF dopaminergic state [19]. It manifests itself somewhat differently during such OFF phases from episodes that occur during the ON phases, one main difference being the duration and severity of the freezing episodes. Any treatment that would alleviate the milder freezing that occurs during an ON period may not automatically be equally effective during an OFF period. This is why we were interested to test our vibrating socks primarily in the OFF dopaminergic state. However, we also felt that it would be a missed opportunity if the vibrating socks’ efficacy were not tested for the milder freezing episodes that may occur during an ON period. Indeed, in most research studies, it is common practice to evaluate devices for alleviation of FOG in both medication states [10, 20].

For the ON dopaminergic state, participants were instructed to continue with their regular dopaminergic medication schedule prior to and during the measurements. For the OFF dopaminergic state, participants were instructed to withhold all dopaminergic medication for at least 12 h prior to the measurement. In people with a deep brain stimulator, stimulation was switched off at least 30 min prior to the OFF state measurement. The order of both sessions was counterbalanced between participants. The gnostic sensibility of the participants was evaluated prior to the start of the first session by applying a Rydel-Seiffer tuning fork to dorsal site of the first metatarsophalangeal joint and the medial malleolus of both feet. Then the vibrating socks were applied and vibrated on a set cadence. Participants were instructed to tap along with the rhythm of the vibration while the researcher felt the vibration with their hands on the vibrating socks. Participants continued with the measurements if they were able to tap along with the vibrating socks. Measurements were conducted at Medisch Spectrum Twente, Enschede or at the Center of Expertise for Parkinson and Movements disorders, Radboudumc, Nijmegen.

**Fig. 1:**
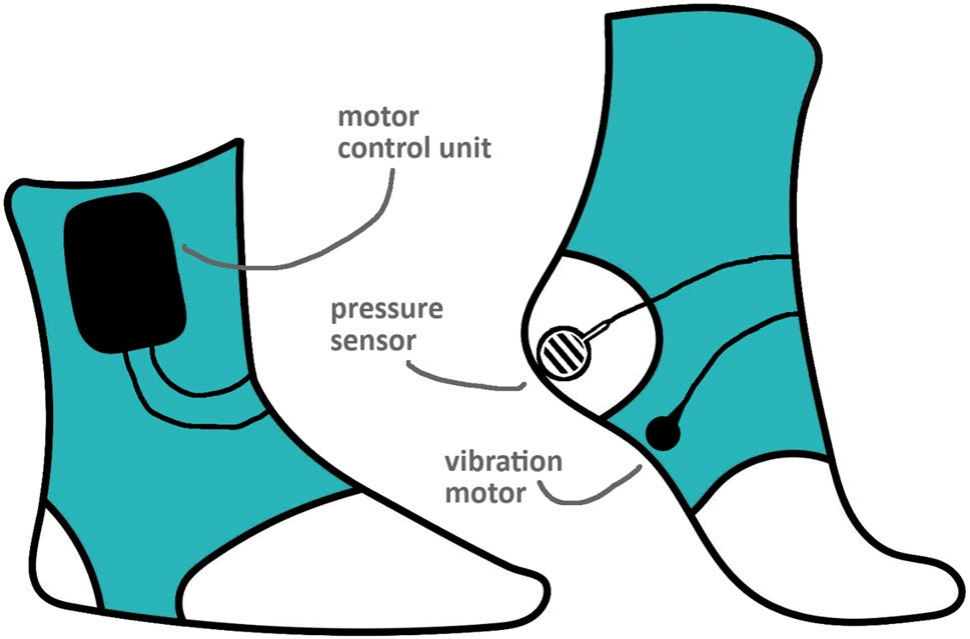
Schematic overview of the vibrating socks, including the motor control unit, pressure sensor (FlexiForce A401 pressure sensor) and vibration motor (Adafruit Mini Motor Disc 1201)

We applied four cueing conditions: (1) closed loop tactile cueing, delivered by the vibrating socks; (2) open loop tactile cueing, delivered by the vibrating socks; (3) auditory cueing (active control); and (4) uncued gait (baseline condition). The order of these four conditions was balanced across subjects, meaning that the cueing condition order was constant within subjects, but varied between subjects to create a balanced dataset. At the start of each experiment, the preferred cadence of the participant was determined per session by walking with a researcher to the beat of a metronome (auditory cue), using the Natural Metronome app [21]. The researcher adjusted the cadence until participants stated they were walking comfortably. Then, patients were familiarized with the open loop and closed loop tactile cueing. During open loop cueing, the socks provided vibrations below the arch of the feet at the preferred cadence frequency, alternating between the left and right foot. Patients were instructed to synchronize their cadence to this alternating pattern, similar to the cadence established using an auditory cue. The prototype of the open loop vibrating socks has been described in previous work by our group [14], containing a flat mini vibration motor (Adafruit Mini Motor Disc 1201) to provide the tactile cueing [14]. The motor disk provides vibrations at 183 Hz and was programmed to vibrate for a duration of 1 s. For the current study, we updated this prototype and added the closed loop functionality. During closed loop cueing, vibration of the socks was activated when the foot of the participant made initial contact with the floor after the swing phase during walking. The vibration was triggered by a pressure sensor (FlexiForce A401 pressure sensor), localized at the sole of both feet of the participant. The socks provided the vibration at 183 Hz until the pressure was released from the sensor. A schematic overview of the vibrating socks can be found in Fig. 1.

During both the ON and OFF sessions, participants performed multiple gait tasks: walking 10 m, turning 360° on the spot (four times to the left, four times to the right), and a gait trajectory. The gait trajectory consisted of two 360° turns while walking (one to the left and one to the right) and walking through a narrow passage. The order of the gait tasks was counterbalanced between subjects. During the gait tasks, participants were videotaped by a Sony HDR-CX240 camera, which allowed for offline rating of FOG. Simultaneously, motion data were collected with a full body suit of the MVN Awinda motion capture system (Xsens, Enschede, the Netherlands), consisting of 17 inertial measurement units. Motion sensor data were collected by Xsens MVN 2020.0.1 software. Participants were instructed to tap one of their feet three times prior to starting the gait task, to facilitate offline synchronization of the motion data with the video data.

The Mini-mental State Examination (MMSE) [22], Frontal Assessment Battery (FAB) [23] and FOG questionnaire [24] were obtained in the ON-state, to document cognitive functioning and freezing severity. The MDS-UPDRS part III and participant satisfaction questionnaire were obtained in both the ON- and OFF state. The participant satisfaction questionnaire consisted of five items rating the performance of the socks, which were rated using a 7-point Likert scale, similar to other work [18]. The five rated items were: (1) the application of the socks (1: very unpleasant–7: very pleasant), (2) the experience of the participant of the closed loop vibration (1: very unpleasant–7: very pleasant), (3) the effect on their walking ability of the closed loop tactile cueing (1: a whole lot worse–7: a whole lot better), (4) the experience of the participant of the open loop vibration (1: very unpleasant–7: very pleasant), (5) the effect on their walking ability of the open loop tactile cueing (1: a whole lot worse–7: a whole lot better).

### Data analysis

The primary outcome measure was percent time frozen, as it is the current gold-standard performance measure of FOG [25]. The percent time frozen was derived from video annotations. Video data were annotated offline in ELAN (ELAN, Version 5.9. Max Planck Institute for Psycholinguistics, Nijmegen) by two independent, trained raters [26]. Annotations were performed in line with the format by Gilat [27], which included annotation of when gait tasks were performed and the occurrence of FOG during these tasks. Percent time frozen was calculated by dividing the duration of the annotated FOG episodes by the duration of the gait tasks. The ELAN annotations were exported to a tab delimited text file. Then, the consensus between the two raters was determined by applying the FOG tool to these text files [28]. The FOG tool determines the differences of start and end of each FOG episode between the two raters and reports the consensus between two raters. Consensus is influenced by two parameters, the tolerance and correction parameter. We set the tolerance to two seconds and the correction parameter to ‘include’, meaning that FOG episodes with a difference of maximum 2 s were included in the FOG episode. The files in which the FOG episodes were in full consensus were exported separately from the files which were not in consensus. The files in which no consensus was reached between FOG episodes were reviewed by a third experienced rater to reach the final FOG rating.

Secondary outcome measures included the number of FOG episodes, derived from the video annotations and spatiotemporal gait parameters, including velocity, step length, cadence and relative durations of the single and double limb support phases, derived from the motion data of the MVN Awinda motion capture system. Extraction of those gait parameters was performed in MATLAB (version 2020b, Mathworks, Inc., Natick, MA, USA) [29].

A post hoc analysis was carried out to identify responders. A participant was defined as a responder to cueing if there was a relative improvement of at least 10% in the percent time frozen in response to that specific cueing modality, as compared to uncued gait. The threshold to define improvement, which was admittedly arbitrarily set at 10%, was set to standardize the amount of improvement of FOG across individuals. This prevents having participants with a minimal improvement being identified as a responders. Further work is needed to define which threshold translates to clinical relevance. Responders were identified in both the ON and OFF state separately. To identify predicting factors of responders, a comparison between the baseline characteristics between responders and non-responders was made.

### Statistical analyses

Statistical analyses were carried out by SPSS (IBM SPSS Statistics for Windows, Version 23.0. Armonk, NY: IBM Corp) [30]. Normality of the distribution of the percent time frozen and amount of FOG episodes was assessed by visual inspection of the corresponding histogram. Subsequently, the effect of the different forms of cueing on both parameters was tested with the Friedman test. Significance level was set at an alpha of 0.05.

## Results

Forty patients were included, of which nine were unable to feel the vibrations of the socks, despite the screening procedure at home. The baseline characteristics of the remaining 31 participants are given in Table 1. All included participants scored above the normal threshold values (≤ 40 years of age 4.5 points; 41–60 years 4.0 points; 61–85 years 3.5 points) for lower extremities of the Rydel-Seiffer tuning fork on an 8-point scale [31]. Their average score was 6.1 (± 1.6) points for the left first metatarsophalangeal joint, 6.1 (± 1.6) points for the right first metatarsophalangeal joint, 6.0 (± 1.2) points for the left medial malleolus and 5.8 (± 1.3) points for the right medial malleolus.

**Table 1:** Baseline characteristics of the vibrating socks study

	Median (25th–75th percentile) or *n* (%)
Age (years)	66 (60–74)
Men	27 (87.1%)
Disease duration (years)	11 (5–14)
Hoehn & Yahr score	2 (2–3)
FOGQ	15 (13–18)
MDS-UPDRS III	
‘ON’ dopaminergic state	38 (29–46)
‘OFF’ dopaminergic state	51 (47–62)
FAB	17 (14–18)
MMSE	28 (26–30)

Depicted as median (25th–75th percentile) or *n* (%)

*FOGQ* freezing of gait questionnaire, *MDS-UPDRS III* Movement Disorder Society–Unified Parkinson’s Disease Rating Scale part III, *FAB* frontal assessment battery, *MMSE* Mini-mental state examination

### Freezing of gait

**Fig. 2:**
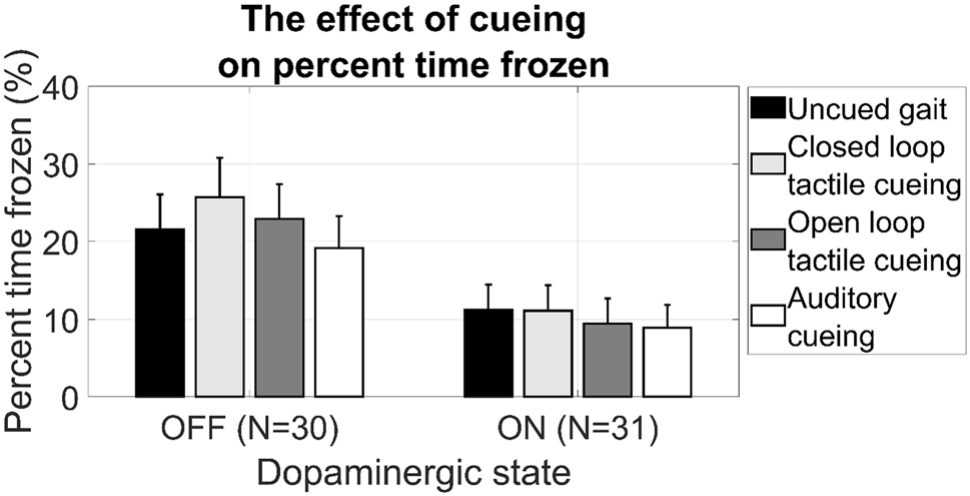
Percent time frozen with cueing and without cueing, in both off and on dopaminergic state. Standard error of the mean (SEM) is indicated by the vertical bars

**Fig. 3:**
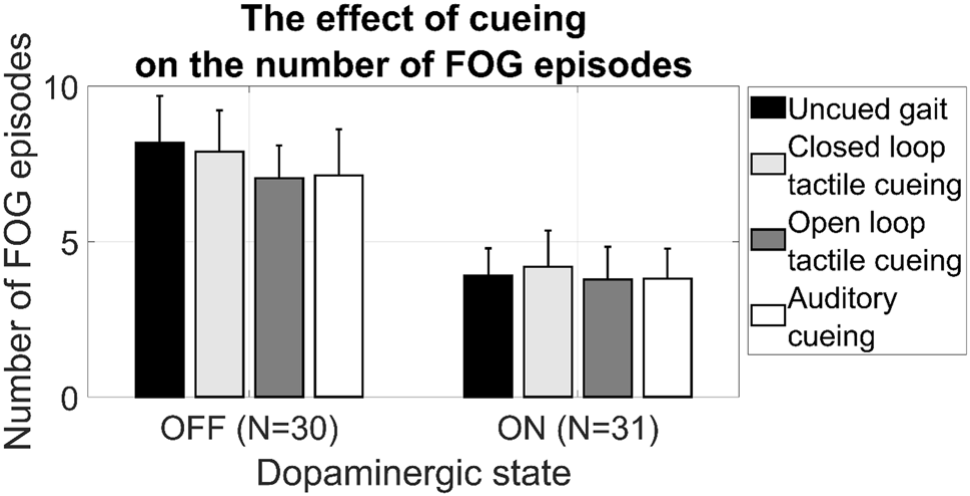
Number of FOG episodes with cueing and without cueing, in both OFF and on medication status. Standard error of the mean (SEM) is indicated by the vertical bars

Twenty-two patients (71%) displayed FOG during one or multiple gait tasks in the ON state and 26 patients (84%) displayed FOG in the OFF state. The average percent time frozen of uncued gait was 11.2% in ON and 21.5% in OFF state. Not all participants were able to complete the full measurement protocol in both the ON (*N* = 4) and OFF state (*N* = 12) due to fatigue, but completed an equal number of tasks during each of the four conditions. At the group level, none of the cueing strategies (closed loop tactile cueing, open loop tactile cueing and auditory cueing) significantly affected the percent time frozen (Fig. 2), neither during ON (*p* = 0.20) nor during OFF (*p* = 0.12). Also, the number of freezing episodes (*p* = 0.72 in OFF; and *p* = 0.77 in ON) as seen in Fig. 3 and the spatiotemporal gait parameters (Table 2) were not significantly affected by any of the cueing strategies.

**Table 2:** Spatiotemporal gait parameters

	Uncued gait Median (IQR)	Closed looptactile cueing Median (IQR)	Open loop tactile cueing Median (IQR)	Auditory cueing Median (IQR)
Velocity (m/s)	0.82 (0.65–1.05)	0.81 (0.66–0.98)	0.84 (0.66–0.96)	0.83 (0.65–1.00)
Step size (m)	0.52 (0.41–0.60)	0.51 (0.41–0.58)	0.50 (0.41–0.59)	0.51 (0.40–0.59)
Cadence (SPM)	108 (104–114)	107 (101–113)	108 (102–114)	107 (97–111)
SLSP	0.88 (0.77–0.94)	0.83 (0.77–0.93)	0.88 (0.77–0.95)	0.83 (0.75–0.93)
DLSP	0.12 (0.06–0.23)	0.17 (0.07–0.23)	0.12 (0.05–0.23)	0.17 (0.07–0.25)

Depicted as median (25th–75th percentile)

*SPM* steps per minute, *SLSP* single leg support phase, *DLSP* double leg support phase

**Fig. 4:**
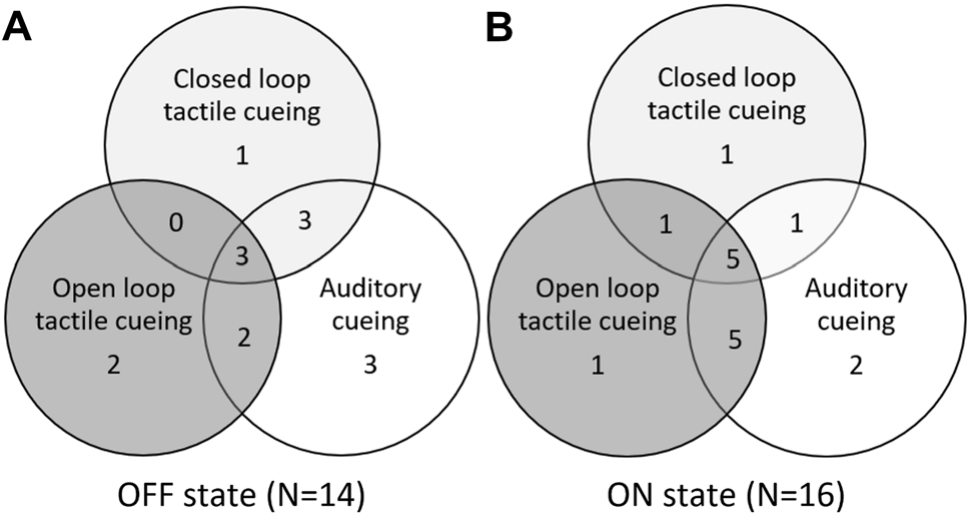
**A** Responders in de OFF dopaminergic state. **B** Responders in de ON dopaminergic state. A responder is defined as a participant who displayed an improvement of at least 10% in the percent time frozen, of that specific cueing modality compared to uncued gait

When looking at the effect of cueing at the individual level, we found that 22 out of 31 subjects responded to cueing (defined as > 10% improvement of FOG using either closed loop tactile cueing, open loop tactile cueing or auditory cueing). Response to the three types of cueing was highly individual: some subjects responded to only one form of cueing but not to the other forms of cueing (see Fig. 4). Also, the within-subject response to cueing differed between the ON and OFF state. Baseline characteristics such as age, years of PD, FAB and MMSE did not differ between responders and non-responders.

### User satisfaction

**Fig. 5:**
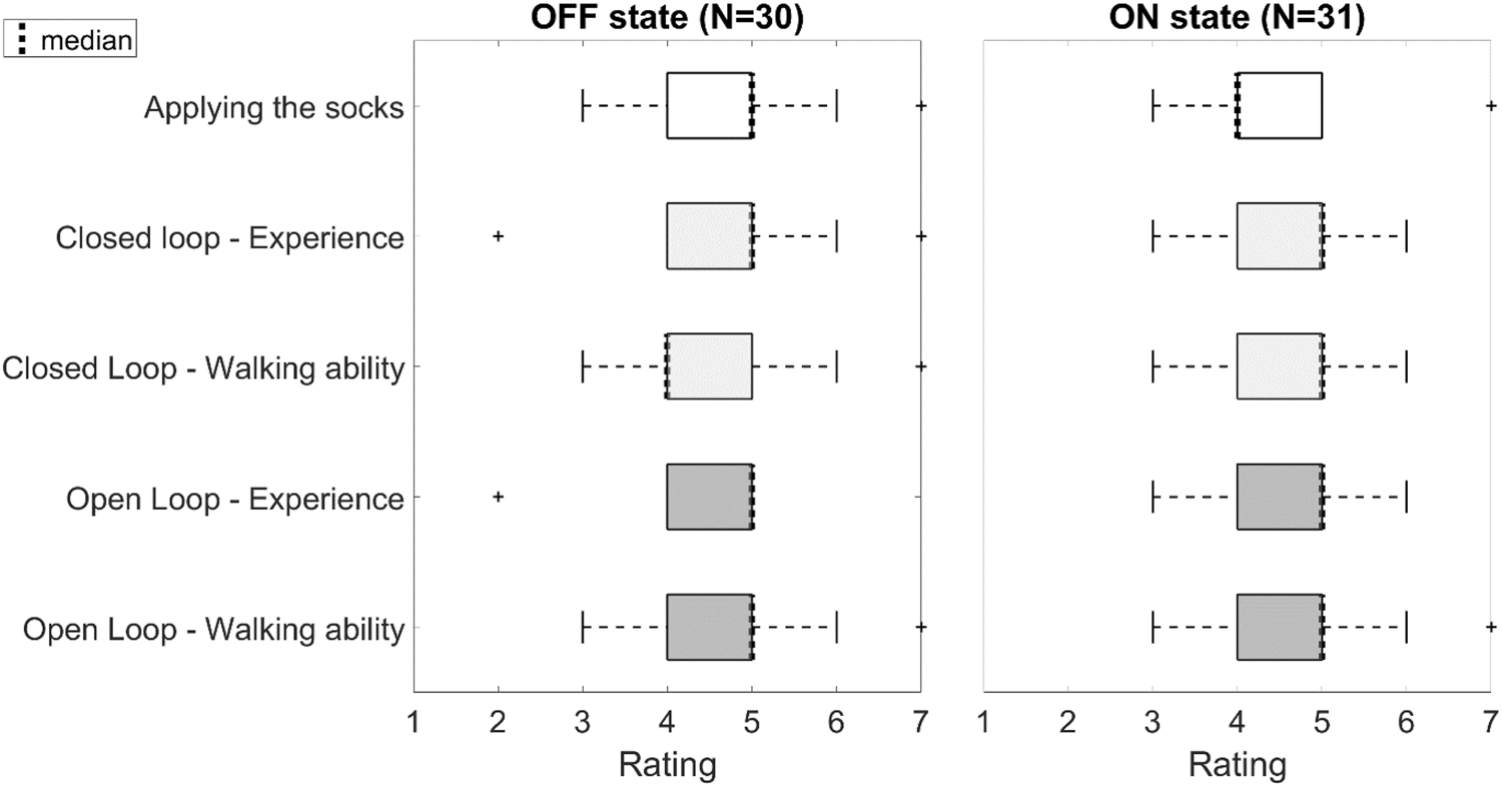
User experience depicted on a 7 point Likert scale. The scale ranged for the application of the socks and user experience, the scaled ranged from 1) very unpleasant to 7) very pleasant. For the walking ability from 1) a whole lot worse to 7) a whole lot better

The user experience was similar for both open loop and closed loop tactile cueing. In the ON- and OFF-dopaminergic state, 75% of the patients rated the application of the socks as neutral to pleasant (Fig. 5). In both dopaminergic states, 75% of the participants experienced the vibration of the socks as neutral or pleasant. Sixty percent of the participants felt their walking ability was improved compared to the uncued gait in the OFF state; 65% felt this was the case in the ON state.

## Discussion

We tested the effect of closed loop tactile cueing, open loop tactile cueing and auditory cueing during various gait tasks in both the ON- and OFF-dopaminergic state. At the group level, we found no effect of cueing on the percent time frozen, the number of FOG episodes or on spatiotemporal parameters. This may be due to the sample size of 31 participants; however, we also found that the effect of cueing was heterogeneous, which may explain these results. Whereas some participants responded well to cueing, others experienced more FOG when cueing was applied. This finding is in line with previous work; not one size fits all with regard to rehabilitation for gait impairments for people with PD [32]. At the individual level, 71% of the participants responded to some form of cueing (> 10% relative reduction of percent time frozen). We found unique responders to each cueing modality which strengthens the previously described importance of an individually tailored, personalized approach when applying compensation strategies in PD [6].

Although proven to be an effective cueing strategy [33, 34], we unexpectedly found no effect of auditory cueing at the group level. This may suggest that only relatively few participants were responsive to cueing. It is known that the effect of external cueing varies greatly among people with PD and even depends on the context in which they are applied [6]. This is reflected by previous studies on tactile cueing; while most found beneficial effects [15, 17, 18, 35], others did not find an effect at the group level, which is in line with our findings [36]. Our findings confirm previous work [36], which evaluated a laser cane, a metronome or a vibrating metronome in 20 PD patients. The vibrating metronome was applied to the pelvis in an open loop matter, similar to our open loop vibrating socks. This study also found that the percentage of freezing episodes did not decrease when comparing cueing to uncued gait at the group level [36]. In contrast, others did find a significant effect of closed loop tactile cueing whilst turning under single and dual tasks conditions [18]. Specifically, these investigators reported a significant decrease in percentage of time spent frozen by closed loop tactile cueing (by as much as 23% during single task conditions, and by 16% while performing dual tasks). Another study also found a significant decrease in the percentage of time spend frozen by tactile cueing in a relatively small group of 10 PD patients [35]. In the latter study, tactile cueing was applied by electrical stimulation alternating between the left and right thigh, fixed to the preferred cadence of the participant. Although the type of stimulus is different (electrical stimulation versus vibration), the pattern of the delivered cue is similar to our open loop version of the vibrating socks. The effect of tactile cueing also has been demonstrated in a smaller group of fifteen participants [17]. These authors found a significant reduction in percentage of time spent frozen by tactile cueing whilst walking and during 180° turns. Tactile cueing was applied to the ankles in an alternating left–right pattern at the preferred cadence of the participant, similar to our open loop tactile cueing [17]. More recent work focused on the effect of visual, auditory and somatosensory cueing on spatiotemporal gait parameters. Twenty participants with PD in the dopaminergic OFF state were evaluated whilst walking. The participants were not impaired by FOG, falls or disabling postural instability. They found a significant in effect for stride length, cadence and velocity. In contrast to our work, all participants received auditory and tactile cueing on the set frequency of 100 beats per minute [37]. Considering the aforementioned results, it is likely that some individual patients, similar to auditory cueing, might actually benefit from tactile cueing [17, 18, 35].

To apply cueing in an individually tailored manner, previous studies searched for patient characteristics to identify responders and non-responders; and found that a better overall cognition is associated with a better response to external cueing [6]. In our study, cognition was assessed using the MMSE and FAB. Most of our participants scored above established cut-off scores for both tests [38, 39]. Therefore, cognitive reserve was not a limiting factor as to why no effect of cueing at the group level was found.

In our study, usability was investigated by a 7-point Likert Scale. The results showed that most participants felt that their walking ability was improved by the vibrating socks (*N* = 20 in the ON state, *N* = 18 in the OFF state). Additionally, 75 percent rated their experience with the vibrating socks as neutral to pleasant. This illustrates an overall positive attitude towards the use of the vibrating socks, which indicates it could potentially be a suitable medical device to use in daily life, when prescribed to appropriately selected individuals.

A limitation of the vibrating sock is that cannot be used in persons with sensory impairments. Despite screening for sensory impairments, 9 out of 40 patients were unable to feel the vibrations of the socks. The gnostic at home test may not have been a good reflection of the participants’ ability to perceive the vibrating socks. In future work and ultimately in daily care, participants should be screened by applying the vibrating socks in a test trial before any actual implementation can take place. This is especially relevant for persons with PD, because the vibration sensitivity of the plantar foot seems decreased compared to age-matched healthy subjects [40]. Specifically, PD patients required a vibration motor to be pushed in their skin further relative to healthy controls to detect the vibration (mean 74.7 µm for PD versus 29.9 µm for healthy controls) [40]. Future technical designs of vibrating socks should take this into consideration, ensuring that the vibration motor is strongly secured in the socks and applied firmly to the skin. An alternative solution for persons with distal sensory decline is to apply the vibration motor at a different (more proximal) body site, as previously has been described [18]. Moving the vibration motor to a different body site may, however, result in a decreased effect, as the cue theoretically would work best to improve strides when applying to the feet. By applying cueing to the feet, the cue is delivered exactly at the location where gait is initiated. A limitation of the present study is that not all participants were able to complete all gait tasks, particularly during the dopaminergic OFF state. However, the same number of gait tasks was collected for all four cueing conditions.

In conclusion, neither auditory nor tactile cueing improved FOG at the group level. However, at the individual level, a relevant proportion of subjects did respond to at least one type of cueing (auditory, open loop tactile cueing or closed loop tactile cueing). This confirms that being responsive to cueing is highly individual and stresses the need for a personalized approach when applying cueing strategies in clinical practice. Therefore, tactile cueing, such as the approach applied here using vibrating socks, could potentially have a place in a personalized healthcare program for people with PD as one of multiple treatment options for FOG. Future research may evaluate the usability of the vibrating socks at home, as a necessary next step before these socks can be considered as a medical device.

## Data Availability

The dataset used in this study is available from the corresponding author upon request.
